# Evaluation of protocols for rRNA depletion-based RNA sequencing of nanogram inputs of mammalian total RNA

**DOI:** 10.1371/journal.pone.0224578

**Published:** 2019-10-31

**Authors:** Simon Haile, Richard D. Corbett, Steve Bilobram, Karen Mungall, Bruno M. Grande, Heather Kirk, Pawan Pandoh, Tina MacLeod, Helen McDonald, Miruna Bala, Robin J. Coope, Richard A. Moore, Andrew J. Mungall, Yongjun Zhao, Ryan D. Morin, Steven J. Jones, Marco A. Marra

**Affiliations:** 1 Canada’s Michael Smith Genome Sciences Centre, BC Cancer, Vancouver, British Columbia, Canada; 2 Department of Molecular Biology and Biochemistry, Simon Fraser University, Burnaby, British Columbia, Canada; 3 Department of Medical Genetics, University of British Columbia, Vancouver, British Columbia, Canada; John Curtin School of Medical Research, AUSTRALIA

## Abstract

Next generation RNA-sequencing (RNA-seq) is a flexible approach that can be applied to a range of applications including global quantification of transcript expression, the characterization of RNA structure such as splicing patterns and profiling of expressed mutations. Many RNA-seq protocols require up to microgram levels of total RNA input amounts to generate high quality data, and thus remain impractical for the limited starting material amounts typically obtained from rare cell populations, such as those from early developmental stages or from laser micro-dissected clinical samples. Here, we present an assessment of the contemporary ribosomal RNA depletion-based protocols, and identify those that are suitable for inputs as low as 1–10 ng of intact total RNA and 100–500 ng of partially degraded RNA from formalin-fixed paraffin-embedded tissues.

## Introduction

Ribosomal RNAs (rRNAs) constitute >90% of the total RNA mass within cells [1–2]. To enhance the sensitivity of RNA-seq to rare mRNA transcripts, methods for either enriching for mRNAs or depletion of rRNAs have been developed. Enrichment for non-rRNA transcripts can be accomplished by strategies targeting their poly(A) tails [3], as most rRNAs are not polyadenylated [4]. However, when applied to Formalin-Fixed Paraffin-Embedded (FFPE) tissues, or otherwise degraded RNA samples, poly(A) enrichment strategies can yield incomplete transcript profiles with a strong bias towards recovery of only the 3’-ends of transcripts. Alternative strategies that address this bias are based on the specific removal of rRNAs [5–7]. These strategies have the added potential advantage of capturing non-ribosomal transcripts that lack polyadenylated tails. A widely adopted commercial kit, illustrative of the type of strategy used in rRNA depletion-based protocols, is the Ribo-Zero Gold kit (Illumina). This protocol uses negative selection of rRNAs via magnetic bead-based affinity purification [5]. New England Biolabs (NEB) has also recently produced an enzyme-based rRNA depletion protocol [6]. Of note, the utility of these commercially available kits is limited to RNA samples from certain species. Alternative protocols that use custom probes, such as the enzymatic probe-directed degradation approach [8] which targets rRNA-derived cDNAs, provide cost-effective options for non-mammalian applications.

Several studies have compared RNA-seq protocols representing the two commercial kits or similar non-commercial protocols [5–15]. The most comprehensive among these studies for nanogram ranges of total RNA inputs considered intact and non-FFPE degraded RNA samples only [15]. Here, we compare two available rRNA depletion kits using intact and FFPE RNA samples across a range of total RNA input amounts. Importantly, we also demonstrate the suitability of FFPE RNA-seq for quantitative gene expression analysis by comparing FFPE-derived data with data derived from a large cohort (n = 39) of matched fresh-frozen tissues.

## Methods and materials

### Samples

Universal Human Reference (UHR) total RNA (Stratagene catalog #740000) was quantified using the RNA 6000 Nano Kit (Agilent, catalog #5067–1511). The External RNA Controls Consortium (ERCC) spike-in mix 1 (Ambion catalog #4456740) was added to UHR total RNA to allow for accuracy and sensitivity assessments. 0.02 μL of the spike-in mix was used per 1 μg UHR total RNA.

Data from 72 samples were analysed for this study. Of these, two of the human FFPE samples were previously reported on [16]. Other FFPE samples, as well as matched fresh-frozen tissue samples, were obtained as part of the National Cancer Institute Office of Cancer Genomic’s Burkitt Lymphoma Genome Sequencing Project (BLGSP) [17]. In general, FFPE tissue samples were ~100 mm^2^ in size (in 2–5 scrolls of 10 μm thickness). Total nucleic acids (DNA and RNA) were extracted from FFPE tissue scrolls using the Agencourt FormaPure (Beckman Coulter) protocol or a combined AllPrep (Qiagen) and High Pure (Roche) protocol, as previously reported [16].

### Ethics statement

Approved by BC Cancer Research Ethics Board, University of British Columbia (Certificate number = H16-02279). Consent was not obtained as data was analysed anonymously.

### Sample preparation for RNA-seq

#### RNase H-based rRNA depletion

The NEB’s RNase H-based rRNA depletion (cat.no. E6310X) was applied to 25–1000 ng total RNA as we previously described [16]. For total RNA inputs in the 1–10 ng range, the volume of the rRNA depletion probe reagent was reduced to 0.5 μL and upstream DNase I treatment was omitted, as the DNase treatment step, which is integral to probe removal in the rRNA depletion kit, was judged to be sufficient to remove residual gDNA contamination.

Following rRNA depletion, cDNA synthesis and library construction steps were performed as described [18]. Thirteen and 15 cycles of PCR were applied for 25–500 ng and 1–10 ng total RNA input amounts, respectively.

#### Ribo-Zero Gold

The Ribo-Zero Gold (Human/Mouse/Rat) kit (cat. no. MRZG126) was purchased from Illumina/Epicentre. rRNA removal and subsequent purification were performed following the manufacturer’s instructions using 2 μL of probe. Subsequent cDNA synthesis and library construction steps were performed as described above for the RNase H-based protocol.

### Sequencing and bioinformatic analyses

RNA-seq libraries were sequenced using paired-end 75 base (PE75) sequencing chemistry on HiSeq 2500 instruments following the manufacturer’s protocols (Illumina). Sequencing data from the BLGSP samples were deposited under phs000527 of the database of Genotypes and Phenotypes (dbGAP) and data from other FFPE samples and UHR were deposited under EGAS00001003849 of European Genome-phenome Archive (EGA).

Alignment-based sequence analysis was performed as described [16, 18]. Briefly, we employed junction-aware BWA [19] alignment to the hg19 reference genome in combination with Ensembl 69 gene models. This process was performed using the JAGuaR junction-aware alignment pipeline [20] which generates BAM [21] files that can be profiled for expression and quality indicators. To control for variable sequence depth, the BAM files were down-sampled and duplicate-marked with sambamba [22] to obtain near-equal numbers of reads suitable for comparing depth dependant results (i.e. duplicate rates and gene detection). Read alignments were subsequently enumerated to generate an expression matrix of sample-by-gene Reads Per Kilobases of transcript per Million mapped reads (RPKM) estimates to allow the evaluation of the similarities in expression profiles between samples. These RPKM values were generated by counting the reads that aligned to annotated gene models and normalizing the counts by the known gene length as well as the total reads aligned to coding regions.

To compare the expression profiles between the matched FFPE and fresh samples, read counts were further corrected for library size using the estimateSizeFactors function in the DESeq2 R package (version 1.14.1) and R version 3.3.2. The corrected read counts were variance-stabilized using the vst function in DESeq2. The pheatmap R package (version 1.0.8) and the 1,000 most variably expressed genes across all samples were used to hierarchically cluster samples using Pearson correlation as the distance metric.

UHR qPCR data for 1000 genes from the MicroArray Quality Control project (GSE5350) [23] were downloaded for comparison to our expression estimates. qPCR values were compared to RPKM values generated with the methods described above. Using samples GSM129638-GSM129641, expression estimates were matched by gene name to allow comparison of our RPKM values and the published qPCR estimates. Each sample was correlated with all four replicate qPCR data sets, from which a median Pearson correlation was calculated.

Structural variant (SV) analysis (i.e. fusion transcript profiling) was performed by combing SV calls generated from multiple methods and combining the results to create consensus calls. The first set of candidate SVs was generated by aligning transcripts generated with Trans-ABySS [24]. These assembled transcripts were created with ABySS1.3.4 [25] employing kmers from k38-k74. Additional candidate SVs were identified by running ChimeraScan [26] and deFuse [27] after which final consensus SVs were reported by MAVIS [28].

ERCC alignments were performed by aligning all reads against the ERCC reference [29] sequences. As these known transcripts do not contain any splicing events alignments were done using BWA [19] mem 0.7.4 with -k set to 40 to ensure specific alignments. As with RPKM calculations for the mammalian data, the read counts were divided by the transcript lengths and total transcript aligned reads before comparing to the known expression values.

## Results and discussion

Previous studies have shown that rRNA depletion protocols are more robust than poly (A)-based protocols for use in applications with lower total RNA amounts or degraded RNA [5–14]. We performed analyses to identify an optimal method for such applications, comparing selected commercially available rRNA depletion protocols.

### Comparison of rRNA depletion protocols for nanogram ranges of intact total RNA input amounts

One class of rRNA depletion protocols involves negative affinity purification, employing magnetic bead-based removal of nuclear and mitochondrial rRNAs using rRNA probes as baits [5]. Another class of protocols involves the enzymatic removal of rRNAs, using RNase H to selectively remove the nuclear and mitochondrial rRNAs that are pre-hybridized to rRNA DNA probes [5–6]. These two classes of protocols are represented commercially in Illumina’s Ribo-Zero Gold (RZG) kit and NEB’s rRNA depletion kit (RNase H), respectively. Here, we sought to compare both protocols across input amounts that ranged from 25–500 ng. For these experiments, we used Universal Human Reference (UHR) total RNA as input. We had initially planned to also include Qiagen’s GeneRead rRNA depletion kit in our comparisons, but discontinued our experiments with it after observing unsatisfactory cDNA yield (S1A Fig). cDNA yields from the RZG and RNase H protocols were comparable for 100–500 ng total RNA input amounts (S1B Fig). RNA-seq libraries were made from 25–500 ng total RNA input amounts to assess rRNA content and other metrics including transcript diversity.

#### rRNA depletion efficiency

**Fig 1:**
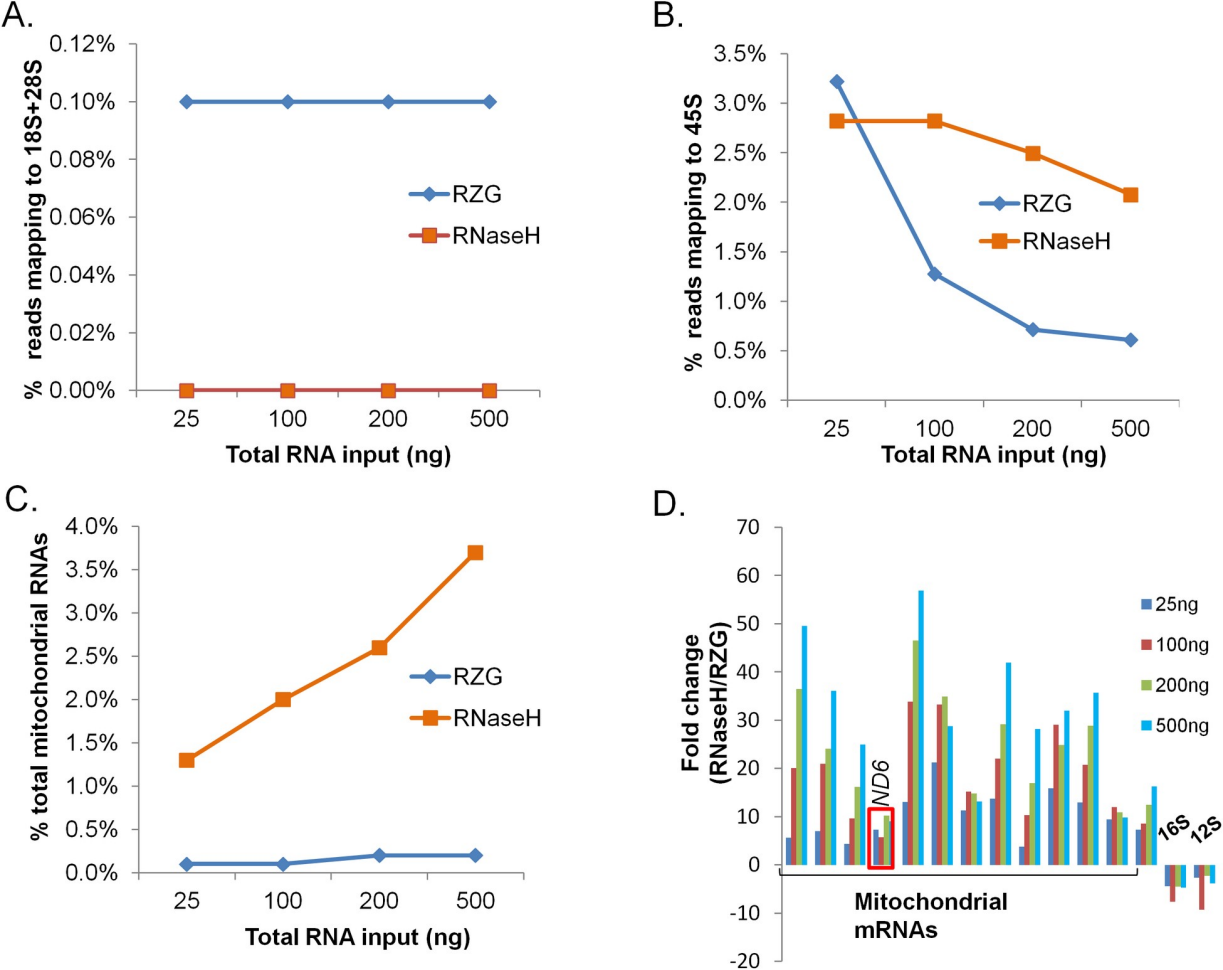
rRNA and mitochondrial transcript content. **Ribo-zero Gold (RZG) vs. NEB RNase H-based rRNA depletion protocol (RNase H).** Input was UHR total RNA at the indicated total RNA input amounts. (A) Reads aligning to 18S and 28S rRNA. (B) Reads aligning to 45S rRNA. (C) Mitochondrial RNA content. (D) Relative levels of each of the mitochondrial mRNAs between the two protocols as compared to the levels of mitochondrial rRNAs.

Both protocols are designed to remove nuclear-encoded rRNAs (18S, 28S, 5S and 5.8S) as well as mitochondrially-encoded rRNAs (12S and 16S). Our analysis revealed that reads mapping to 18S and 28S were observed to be ~ 0.1% of total reads for the RZG protocol, with no such reads detected in data from the RNase H protocol (Fig 1A). No reads from either protocol aligned to 5S and 5.8S sequences. We also quantified reads mapping to the 45S precursor rRNA, which is comprised of 18S, 5.8S, 28S and internal and external rRNA spacer regions. Both protocols generated 0.5–3.5% of reads mapping to 45S (Fig 1B). In contrast to reads mapping to the mature rRNA species, reads mapping to 45S were ~2% higher when using the RNase H protocol for all total RNA input amounts except 25 ng, where the proportion of 45S reads were comparable (Fig 1B). One possible reason for the discrepancy in the relative abundance of precursor versus mature rRNA reads may be that the RZG protocol depletes the entire precursor rRNA, as it is a rRNA probe-tagged affinity purification-based protocol. In contrast, the RNase H protocol, being a probe-targeted enzymatic degradation approach, is expected to only deplete regions of RNAs hybridizing to the probes to form RNA:DNA hybrids. These probes do not blanket the entire precursor 45S RNA, instead specifically targeting mature rRNAs.

The proportion of mitochondrial transcripts out of total number of reads, including mitochondrial rRNAs and mitochondrial mRNAs, was 1–4% for the RNase H protocol and was positively correlated with RNA input amounts, whereas the RZG data displayed <0.3% for all input amounts (Fig 1C). The higher proportional mitochondrial RNA content seen in the RNase H data was not due to its inefficiency in removing the targeted mitochondrial rRNAs (12S and 16S rRNAs). Instead, the RNase H protocol was more efficient in that regard for all input amounts (Fig 1D). In contrast, the RZG protocol resulted in a significantly lower proportion of non-ribosomal mitochondrial transcripts (*p*<0.05) suggesting an off-target depletion effect for this protocol (Fig 1D); i.e. non-target sequences that did not hybridize to the probe sequences were depleted when using the RZG protocol.

One explanation for this may be found in the polycistronic transcription of the mitochondrial genome, which forms a long precursor RNA that is subsequently processed to yield mature rRNAs, tRNAs and mRNAs. It is conceivable that the RZG protocol, unlike the RNaseH protocol, depletes the precursor mitochondrial RNAs in addition to the mature rRNAs, leading to a general decrease in abundance of all mitochondrially encoded transcripts. However, this is cannot explain the depletion of ND6 RNA, which is encoded by the light DNA strand separately from the heavy strand that encodes mitochondrial rRNAs, is also partially depleted in the RZG libraries (Fig 1D).

#### Diversity and expression correlation across input amounts

**Fig 2:**
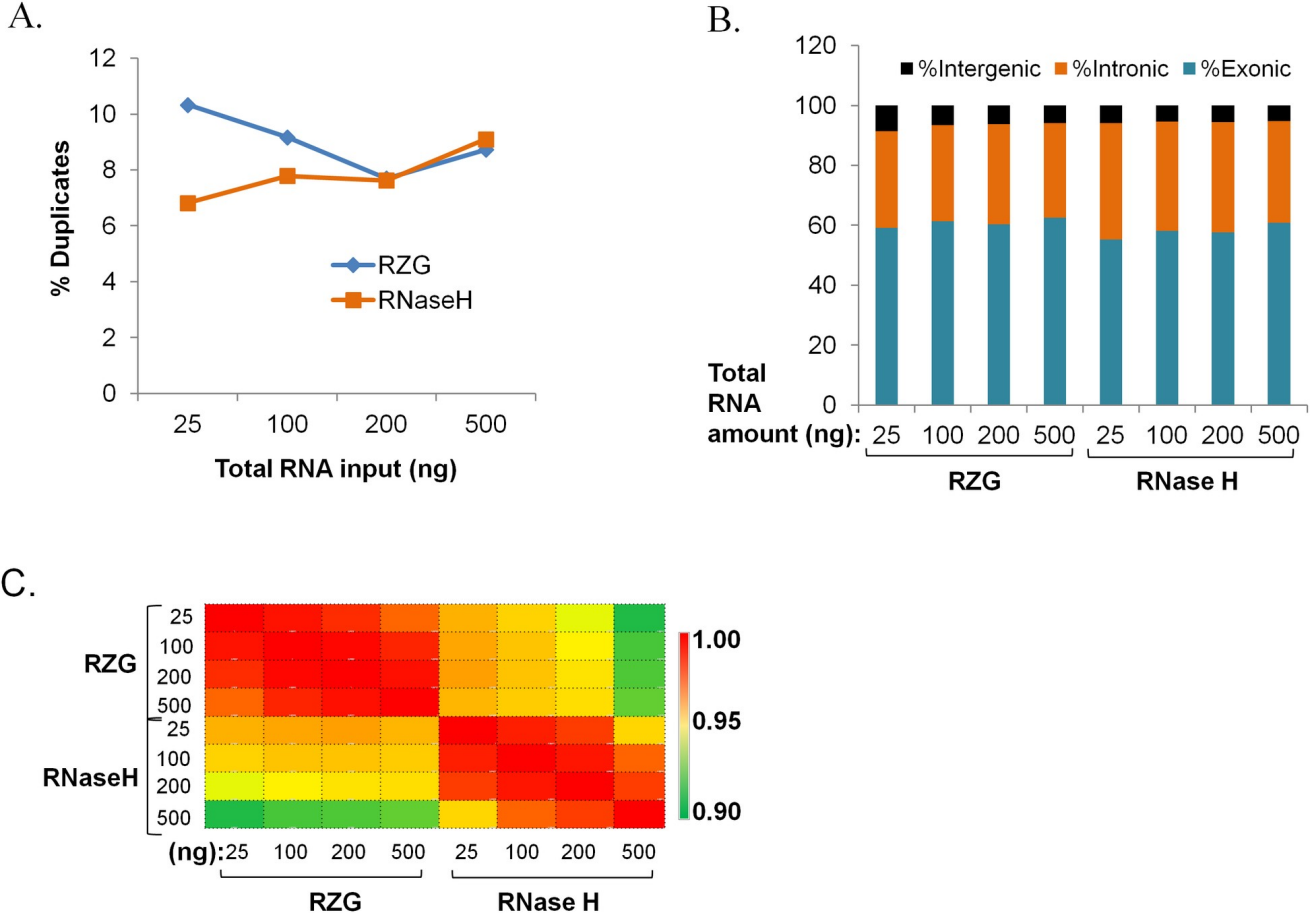
Diversity, regional mapping and expression correlations. Ribo-zero Gold (RZG) vs NEB RNase H-based rRNA depletion protocol (RNase H). Input was UHR total RNA at the indicated total RNA input amounts. (A) Proportions of duplicate reads. (B) Proportions of exonic, intronic and intergenic reads. (C) Expression correlations across RNA input amounts. Pearson’s correlation coefficient was calculated pair-wise for all transcripts.

We compared the duplicate read prevalence between libraries generated using both rRNA depletion protocols and found that the RNase H protocol yielded libraries that had lower duplicate rates for 25 and 100 ng input amounts (Fig 2A). The comparable duplicate rates across these input amounts appears to be unique to rRNA depletion protocols; poly (A)-based libraries do show differences in this input range [18]. The two rRNA depletion protocols yielded comparable proportions (>30%) of reads mapping to intronic regions (Fig 2B), which we expected to recover using both protocols. In contrast, we previously observed that poly (A)-based libraries from similar input amounts of UHR total RNA yielded 7–9% of reads mapping to intronic regions [18].

Expression of >24,000 UHR genes was highly correlated across varying input amounts in both protocols (*r*>0.95) with the RZG showing slightly higher correlation of expression between the lowest and highest input amounts (0.98 for RZG vs. 0.96 for RNase H based on the 500 ng vs. 25 ng total RNA input comparison) (Fig 2C).

#### Validation of expression accuracy and dynamics

**Fig 3:**
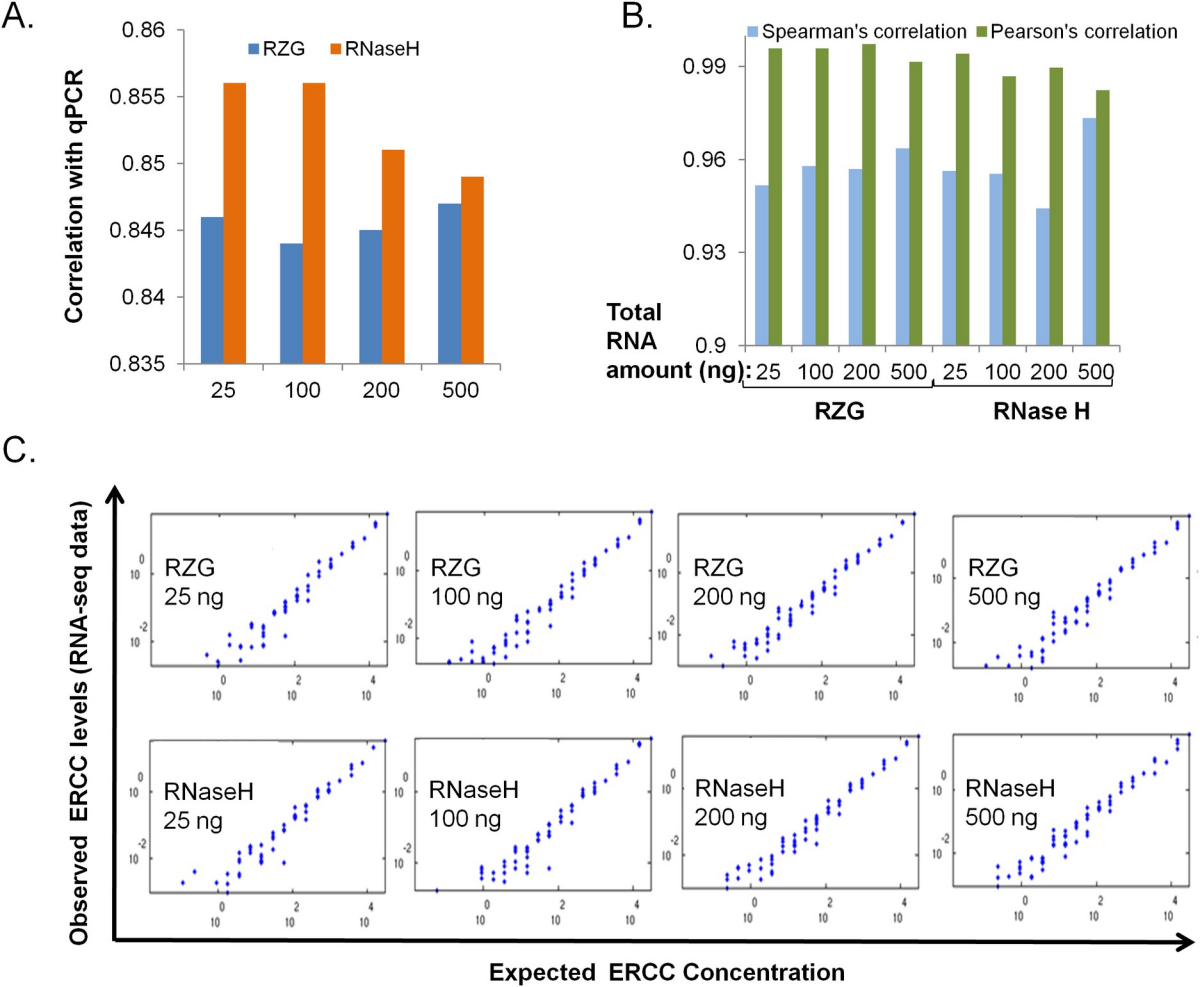
Validation of expression quantification accuracy. Ribo-zero Gold (RZG) vs NEB RNase H-based rRNA depletion protocol (RNase H). Input was UHR total RNA at the indicated total RNA input amounts. (A) qPCR data for ~1,000 mRNAs [23] compared to RNA-seq data. (B) Correlation of observed versus expected ERCC spike-in levels. (C) Log-log plots of observed versus expected ERCC RNAs. Blue dots represent amounts of individual spike-in RNAs, the number of which is variable between libraries depending on the detection sensitivity of the protocol.

UHR transcript expression levels of 1,000 genes were previously quantified using TaqMan qPCR assays [23]. We compared the expression values derived from this data set with those of the RNA-seq libraries that were generated using the RNase H and RZG protocols. As shown in Fig 3A, all of the RNA-seq libraries displayed *r*>0.84, with the RNase H libraries showing slightly higher correlation values (*p* = 0.0074).

Further assessment of the accuracy of quantitative gene expression was obtained by exploiting the ERCC spike-in RNA mix that has been established as a standard for RNA-seq platform evaluation [29–30]. This mix contains 92 synthetic RNAs of known and diverse lengths and sequences at predefined varying concentrations, which we added to the UHR RNA prior to performing rRNA depletion. We compared the performance of both protocols, across the range of inputs, in detecting ERCC RNAs and showed that both protocols allowed the detection of 63–76% of the ERCC RNAs. To compare the sensitivity of the two protocols in detecting ERCC RNAs, we determined the concentration of ERCC RNA concentration that allowed a 50% probability of detection using a logistical regression approach that was described previously [31] (S2 Fig). Such assessment did not reveal a significant difference in the sensitivity of detection between the two protocols across the various total RNA input levels (*p* = 0.7884).

For comparison of the accuracy of the measurement ERCC RNA levels, we compared the observed yield to the expected yield of ERCCs and found that both the RZG and RNase H protocols resulted in high ERCC correlation values (r>0.98) with the RZG data exhibiting slightly higher correlation values (*p* = 0.049) (**Fig 3B and 3C**).

### Comparison of rRNA depletion protocols for nanogram amounts of total RNA derived from FFPE tissues

**Fig 4:**
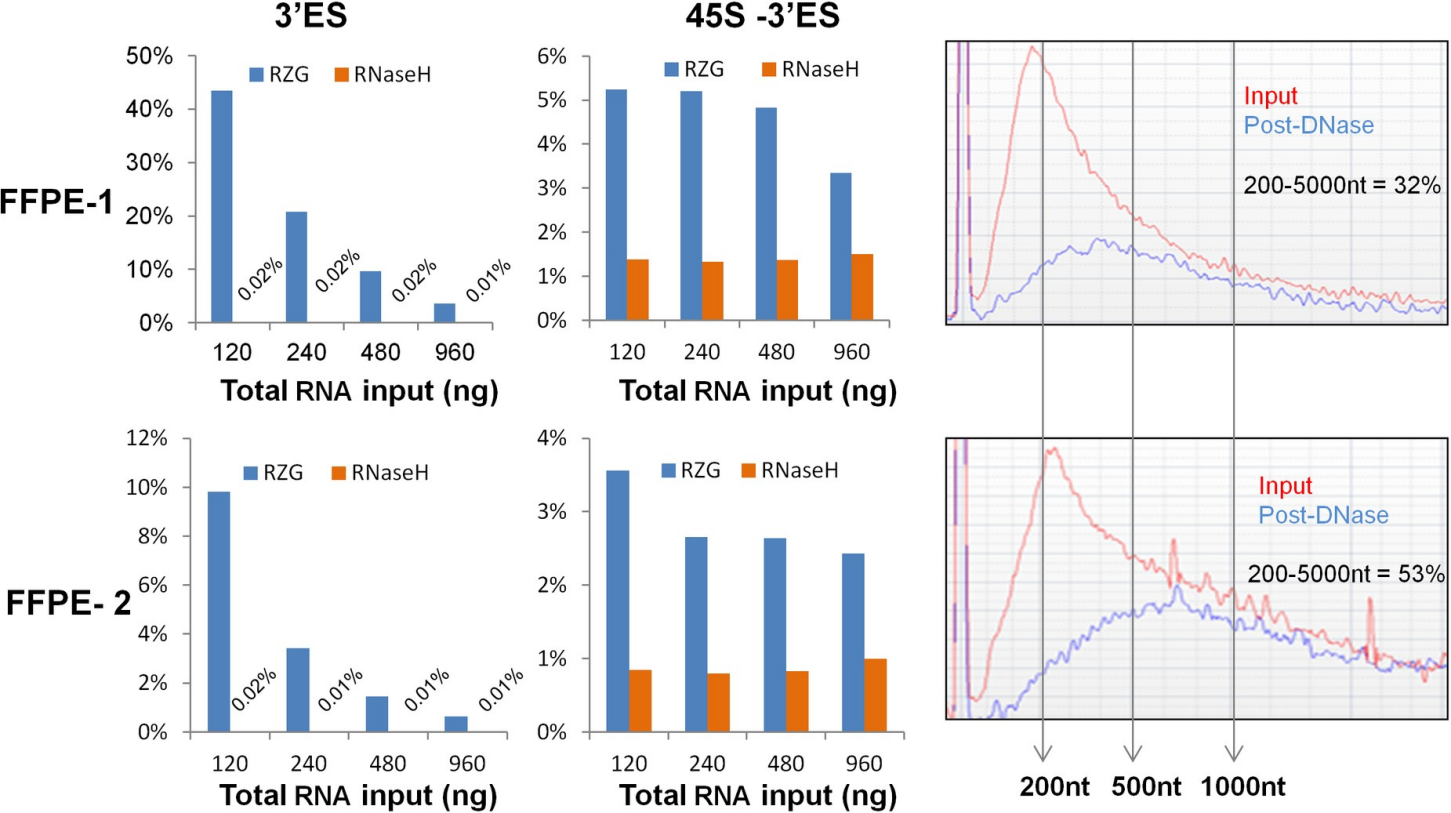
rRNA content comparisons using FFPE samples. **Ribo-zero Gold (RZG) vs NEB RNase H-based rRNA depletion protocol (RNase H).** Input was FFPE total RNA at the indicated total RNA input amounts. The two samples, FFPE-1 and FFPE-2, are described in the text. Reads mapping to the 3’-external spacer (3’ES) are shown in the left panels (top and bottom) and reads mapping to other regions of the 45S precursor RNA are shown in the middle panels (top and bottom). RNA size profiles from Agilent RNA Nano assays are shown in the right panels. In red are the profiles for the RNA input before DNase I treatment (Input) and in blue are profiles for RNA after DNase I treatment (Post-DNase). Vertical arrows delineate indicated sizes in nucleotides (nt) and the proportions of fragments between 200 and 5000 nt are indicated in the insets.

We next compared the performance of the two protocols using formalin fixed paraffin embedded (FFPE) RNA as input, such as what one might purify from clinically obtained patient samples. For this, we used two FFPE blocks; one that was prepared 19 years prior to RNA extraction from a tumor sample of a patient with the diagnosis of follicular small cleaved—grade1 lymphoma (FFPE-1). The other block was prepared 5 years prior to RNA extraction from a tumor sample of a patient diagnosed with diffuse large B-cell lymphoma (FFPE-2). We used total RNA input amounts ranging from 120 ng to 960 ng. cDNA yields from FFPE-1, using the RZG protocol with 13 cycles of PCR, were relatively high, ranging from 8–80 nM, which was 4 to 8-fold higher than yields obtained using the RNase H protocol (S3A Fig). For FFPE-2, the library yield was higher than that obtained for FFPE-1 and the library yield achieved using the RZG protocol was 1.5 to 3.3-fold higher than that obtained using the RNase H protocol (S3A and S3B Fig). The differences in library yield may reflect the quality of the respective RNA used to make the libraries (Fig 4; right panel), which may be related to the age of the clinical materials from which the RNA was extracted.

All libraries were pooled and sequenced in a single MiSeq run, resulting in at least one million paired end reads per library. Analysis of these data revealed that the proportion of reads classified as “intergenic” were higher at lower input amounts in the libraries prepared using the RZG protocol **(**S4A Fig**).** For both protocols, the percentage of duplicate reads strongly and positively correlated with the percentage of ‘intergenic” reads (*r* = 0.96–1.00) (S4B Fig**).** Up to 40% of the apparently intergenic duplicate reads actually aligned to the 3’-end of the chromosomally non-localised contig, GPL220.1 of build GRCh38 (S5 Fig). This contig harbors the 3’-external spacer (3’-ES) region of the 45S precursor rRNA (down-stream of the 28S sequence), which is not included in the ensembl annotation, resulting in our mis-classification of such reads as “intergenic”. We enumerated the reads that mapped to the 3’-ES region in all libraries and found that the libraries generated using the RZG protocol had a much higher proportion (up to 2000-fold) of such reads, especially at lower input amounts (Fig 4; left panels), mirroring the trend observed at the library yield and duplicate read levels. These results are in contrast to the data obtained for UHR libraries, in which the difference between the two protocols in terms of the proportion of reads mapping to the 3’-ES region was relatively small (0.1% to 2.4%; S6 Fig). We note that the 3’-ES read content was generally higher for FFPE-1 (Fig 4; left panels), which had lower quality RNA (Fig 4; right panels).

Although NEB (the manufacturer of the RNase H kit) explicitly states that the mature nuclear and mitochondrial-encoded rRNAs are targeted for depletion, they do not state in their product description that the kit includes probes targeting the 3’-ES. Our results are compatible with the notion that the 3’-ES region is, in fact, targeted for removal in the RNase H protocol but not in the RZG protocol. Consistent with our results, another study [5] that employed the RNase H protocol (but not the NEB reagents) reported that the RNase H protocol was better at removing rRNAs (including 3’-ES) than the RZG protocol, with the percentage of 3’-ES reads being 46% for RZG and 0.06% for RNase H. However, this study used only one FFPE sample at only one total RNA input amount (1,000 ng). At a comparable input amount (960 ng), we observed 3.6% 3’-ES reads for RZG and 0.01% for RNase H, a difference that perhaps reflects input RNA quality, the exact rRNA depletion protocol applied, or other differences between the two protocols.

### Comparison of fresh-frozen and FFPE libraries prepared using the RNase H-based rRNA depletion protocol

**Fig 5:**
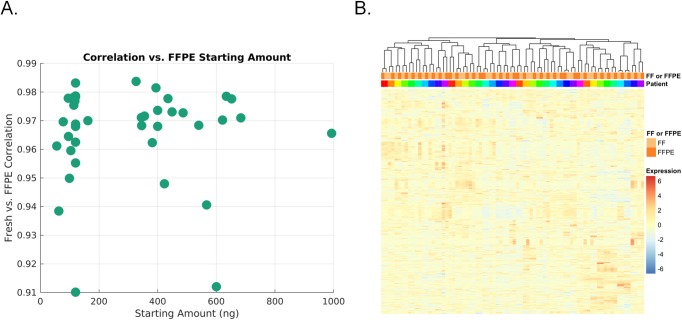
Expression correlation and hierarchical clustering of data from matched fresh-frozen and FFPE- derived samples (n = 39). (A) Pearson’s correlation of transcript levels between fresh-frozen and FFPE samples (Y-axis) for various total RNA input amounts (X-axis). (B) Hierarchical clustering. Variance-stabilized expression values for 1,000 genes whose expression was most variable were chosen for clustering. Samples were hierarchically clustered based on inter-sample Pearson correlation values. The results indicate that FFPE preparation of samples does not result in a dominant batch effect that occludes the biological source of the material (i.e., the patient’s tumour).

Given the improved performance of the RNase H protocol compared to the RZG protocol in our preliminary FFPE analyses, we next evaluated the FFPE RNase H protocol for use at scale, comparing the data with those obtained from matching fresh frozen (FF) samples from the same patients. We used tumor samples from 39 patients diagnosed with Burkitt’s lymphoma, obtained as part of the Burkitt Lymphoma Genome Sequencing Project (BLGSP) [17]. Input amounts ranged from 120 ng to 1000 ng DNase I-treated RNA for FFPE RNA, and 200–300 ng for fresh RNAs. Libraries from fresh frozen samples were generated using a protocol we described previously [16, 18]. We found that the abundance of duplicate sequencing reads was comparable between FFPE and FF libraries and was correlated with the amount of input used (S7A Fig). Read coverage of annotated transcripts tended to be less uniform in the FFPE libraries compared to the FF tissue libraries (S7B Fig) and exon to intron ratios were lower for FFPE (S7C Fig), consistent with previous reports for libraries prepared using the RNase H [14] or the RZG protocols [32]. Despite these differences, the expression correlation between matched FFPE and FF samples was high (*r =* 0.923–0.994) (Fig 5A) and all libraries clustered according to patient source and not based on whether they were derived from FFPE or FF tissue (Fig 5B).

Thus, using a substantial sample cohort of matched FFPE and FF tissue data, our analyses indicate that the RNAse H protocol for rRNA depletion exhibits improved performance for expression analysis of FFPE samples, such as those typically obtained for clinical purposes, which typically suffer from partially degraded RNA. Given the better performance of the RNaseH protocol on FFPE samples, we focused on this protocol for further assessment as described below.

### Sensitivity of fusion RNA detection

**Fig 6:**
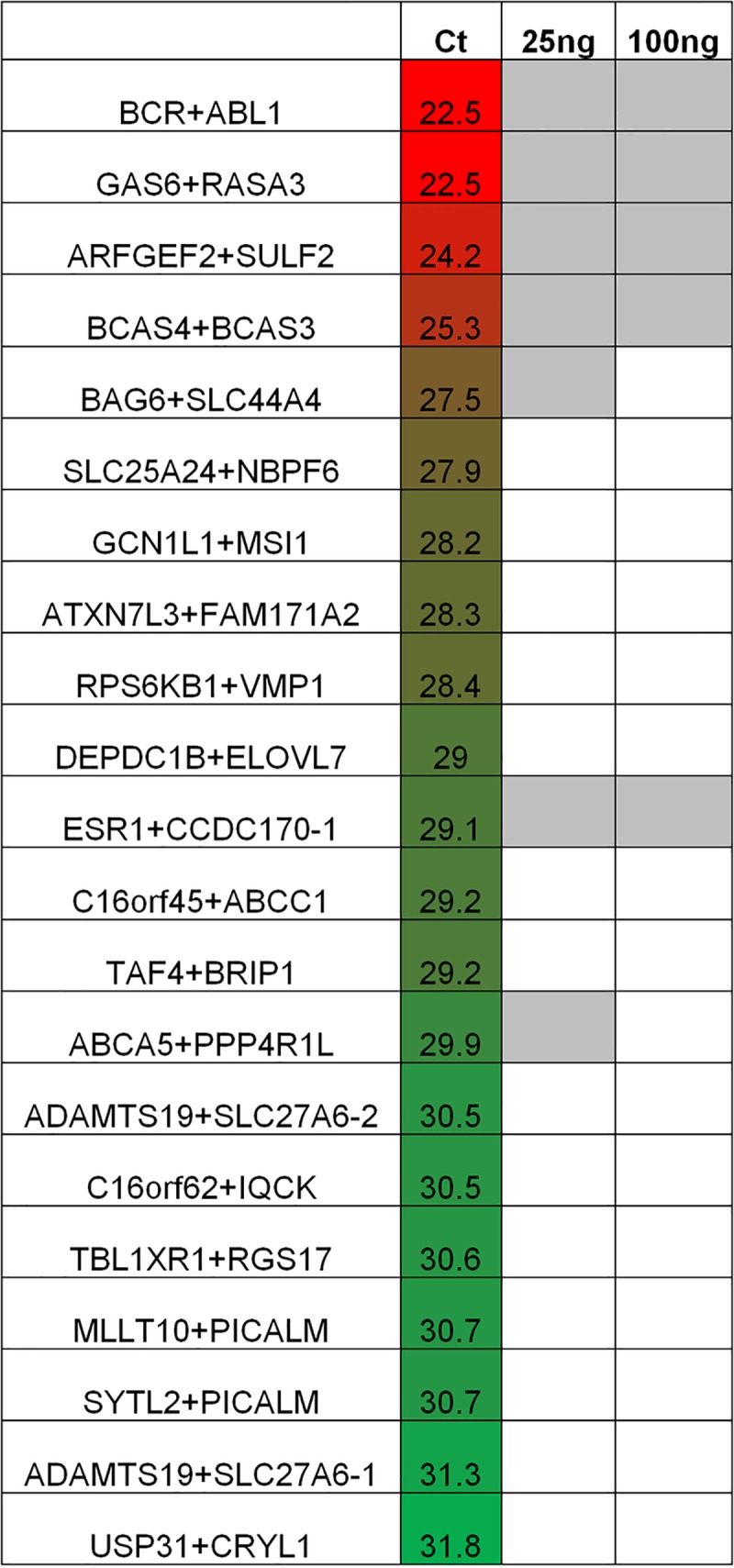
Analysis of fusion transcripts in the RNase H protocol. 25 ng and 100 ng UHR total RNA input libraries were evaluated for the detection of events that were previously validated using qPCR [33]. Filled gray boxes indicate that events were positively identified. qPCR cycle threshold (Ct) data are from [33].

In addition to quantification of canonical transcripts, RNA-seq has been used for assessment of fusion transcripts arising from rearrangements in the genome that affect coding sequences (e.g. [33–34]). To evaluate the RNase H protocol for detection of gene fusions, we sequenced libraries from 25 and 100 ng UHR total RNA input amounts to a depth of ~300 million paired-end reads in one lane of an Illumina HiSeq 2000 instrument. After applying a combination of assembly and various alignment-based fusion detection methods, data were evaluated for the presence of known UHR gene fusion and rearrangement events that were previously validated using qPCR [33]. Of the 21 different events evaluated, 7 were positively identified in the 25 ng and/or 100 ng libraries (Fig 6). The majority of the events that were not supported in the two libraries were only border-line detected by qPCR [33]) (Fig 6).

### Evaluation of the RNase H-based rRNA depletion protocol for lower nanogram range of intact total RNA input amounts

**Fig 7:**
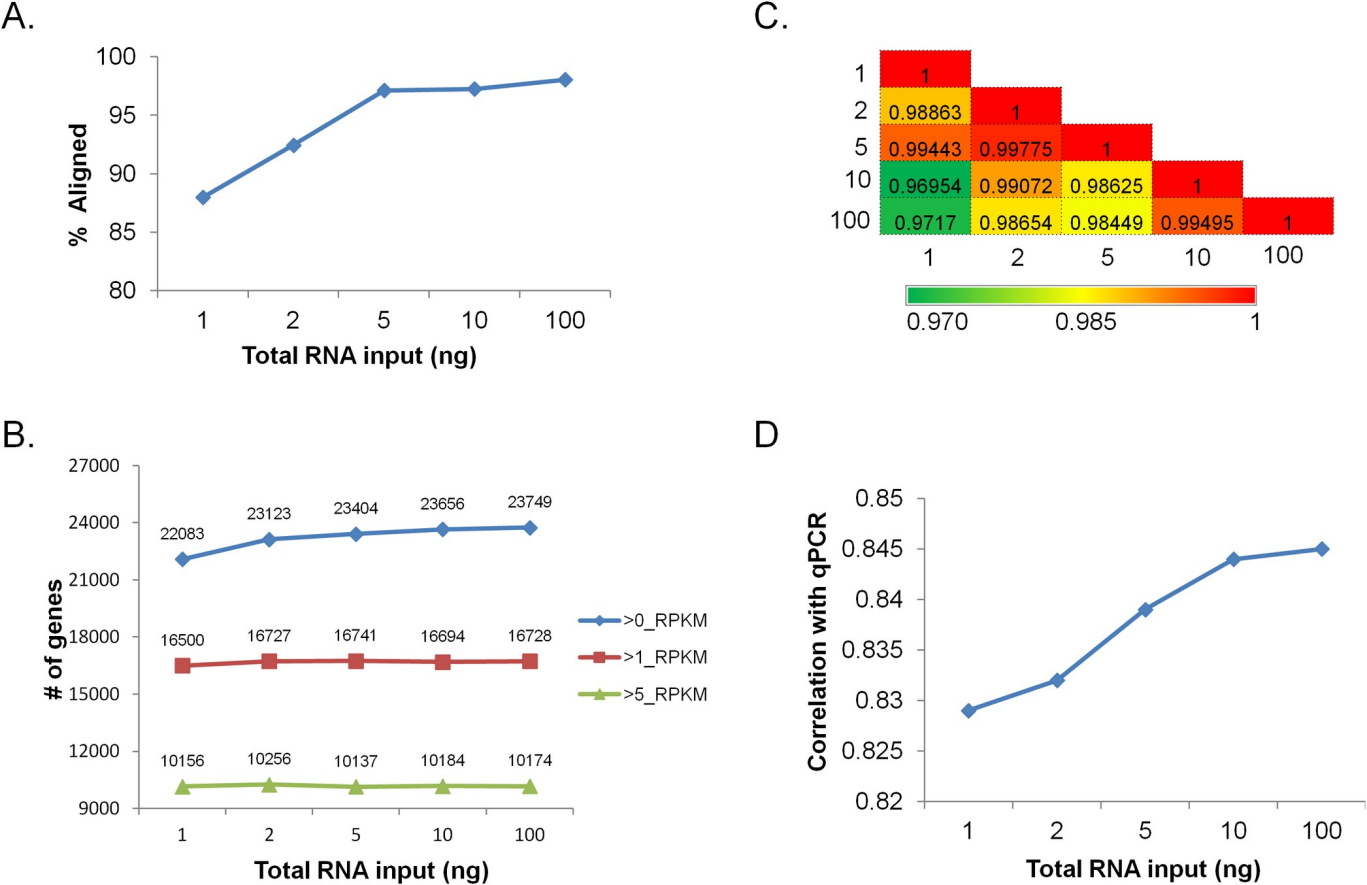
Effects of lowering input amounts using the RNase H protocol. **The input was UHR total RNA at indicated total RNA input amounts.** (A) Proportion of aligned reads achieved using 1–100 ng of total RNA input. (B) Genes identified as a function of input amount. Blue indicates genes identified with greater than 0 RPKM values; red indicates genes identified with greater than 1 RPKM values, and green indicate genes identified with greater than 5 RPKM values (indicative of more abundant transcripts). (C) Expression correlations across UHR RNA input amounts, indicated by numbers on both axes. Pearson’s correlation coefficient was calculated pair-wise for all transcripts. (D) Orthogonal validation of expression accuracy. Previous qPCR data for ~1000 mRNAs was compared with RNA-seq data [23].

We next wanted to determine the suitability of the RNase H protocol for 1 ng to 100 ng intact total RNA input amounts. Proportions of aligning reads for the lower total RNA input amounts (1–2 ng) libraries (87–95%) were lower than for those obtained for the higher input (5–500 ng) libraries (94–97%) (Fig 7A**)**. Library diversity was also lower for the lower input libraries (Fig 7B**)**. Despite these differences, correlation of levels of UHR transcripts among the different input libraries was high (*r* = 0.969–0.997), although there was a trend for the lower input libraries to show slightly lower correlations with the higher input libraries (Fig 7C). Similar input dependency of correlation values was observed when UHR data was compared with qPCR data (Fig 7D).

Overall, the data described above indicate that the RNase H-based and Ribozero Gold rRNA depletion protocols can be used for as little as 10 ng intact total RNA input amount without significant loss of sequencing data quality. We further show that, using the RNase H protocol, libraries of acceptable data quality can be generated from as low as 1 ng of intact total RNA, and that the RNAse H protocol appears to have generally superior performance for the analysis of partially degraded RNA.

## Supporting information

S1 Fig cDNA yield comparisons. (TIF)

S2 Fig Sensitivity of ERCC detection. (TIF)

S3 Fig Comparisons of library yield using FFPE samples. (TIF)

S4 Fig Comparisons of intergenic and duplicate reads using FFPE samples. (TIF)

S5 Fig Demonstration of the levels of reads mapping to the 45S 3’-ES region using IGV in FFPE samples. (TIF)

S6 Fig Proportion of the reads mapping to the 45S 3’-ES region in UHR. (TIF)

S7 Fig Comparisons of sequencing quality between FFPE and FF libraries. (TIF)

## Abbreviations

RNA-seq: RNA sequencing
rRNA: Ribosomal RNA
ERCC: External RNA Controls Consortium
FFPE: Formalin-Fixed Paraffin-Embedded
FF: Fresh-frozen
RZG: Ribo-Zero Gold
UHR: Universal Human Reference
