# Supplementary figures and images for: Evaluation of protocols for rRNA depletion-based RNA sequencing of nanogram inputs of mammalian total RNA
Source: PLoS One. 2019 Oct 31;14(10):e0224578. doi: 10.1371/journal.pone.0224578 (PMC6822755; doi:10.1371/journal.pone.0224578)

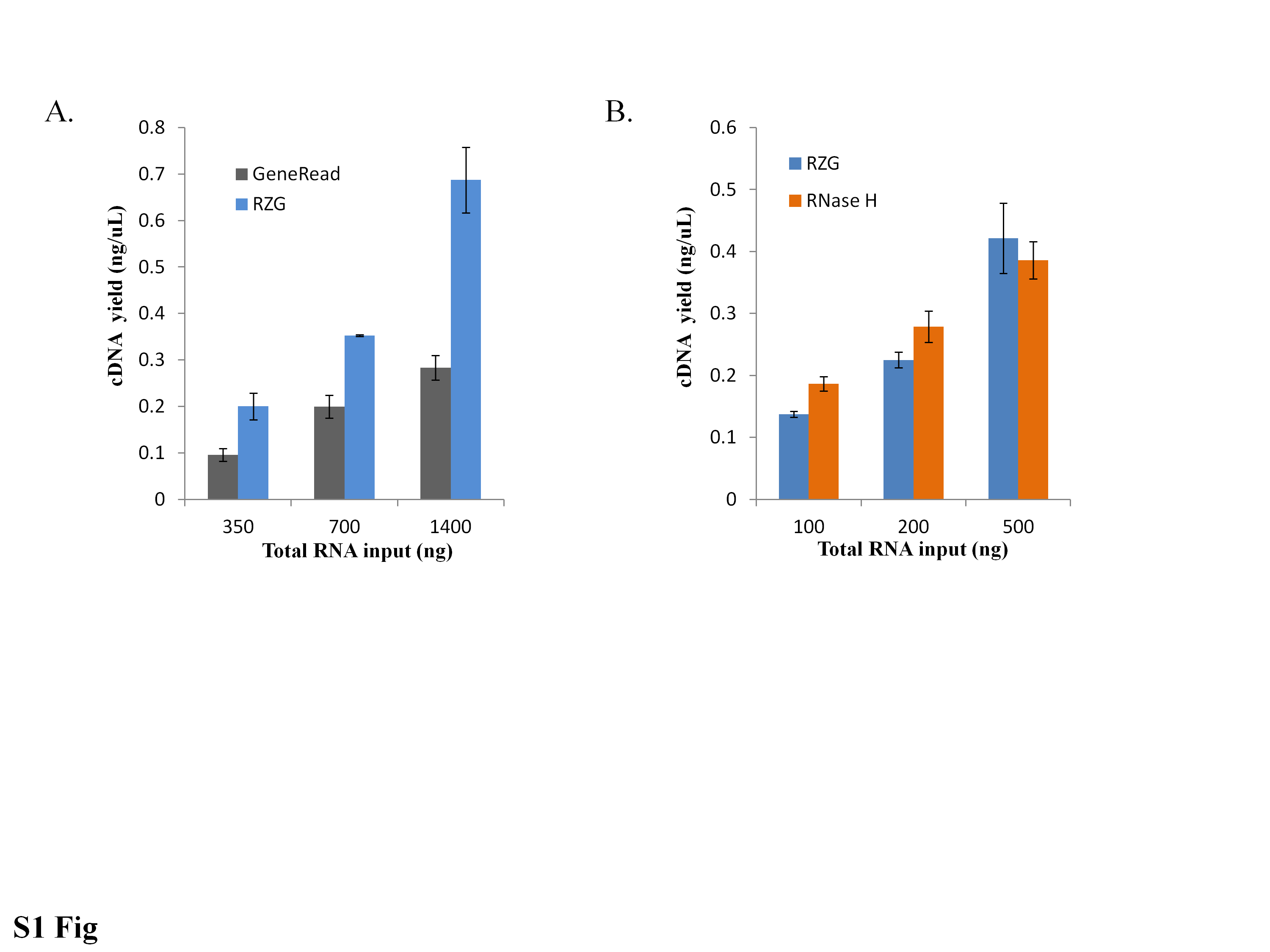

Supplement: S1 Fig — (TIF) [file pone.0224578.s001.TIF]

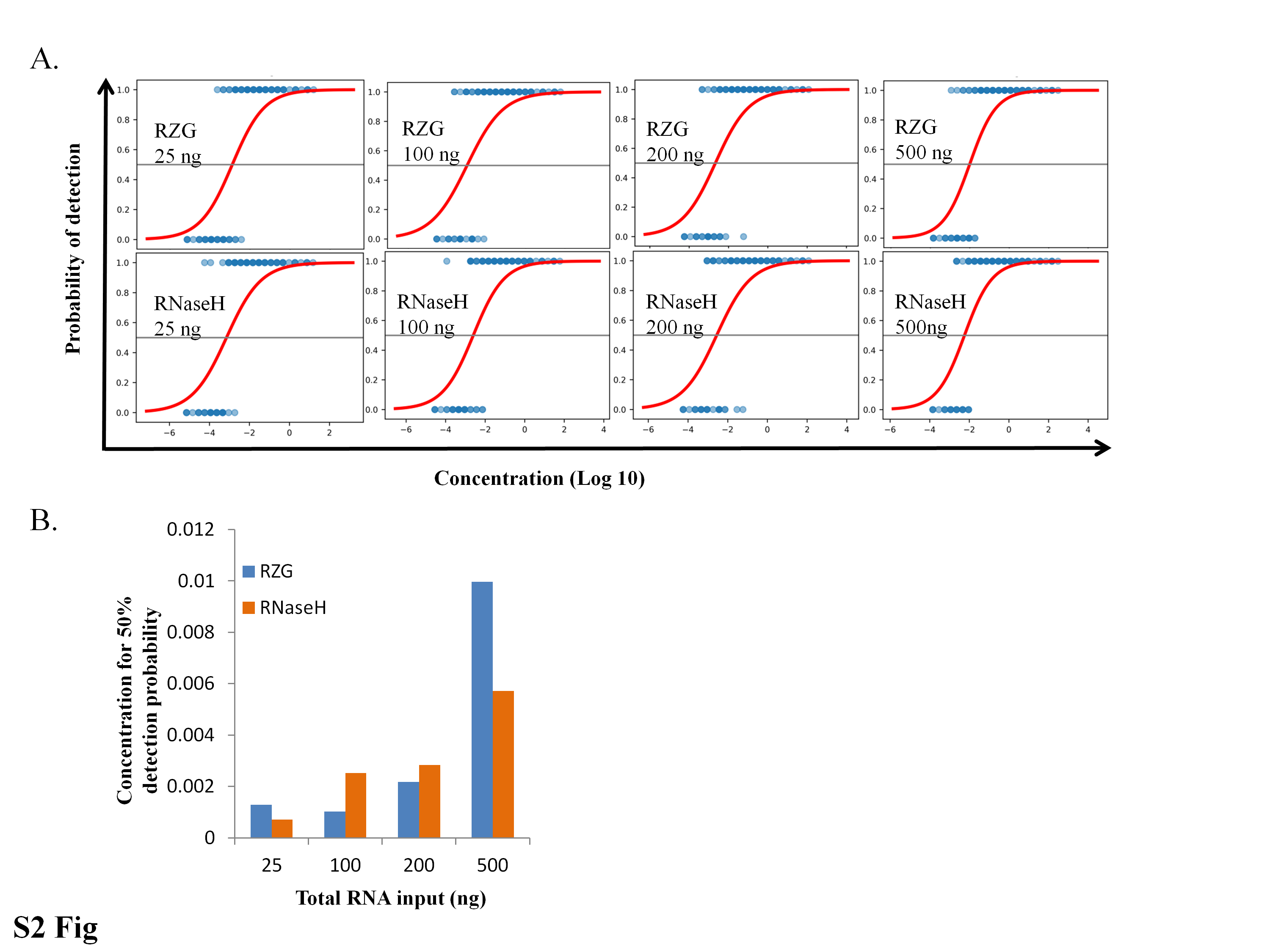

Supplement: S2 Fig — (TIF) [file pone.0224578.s002.TIF]

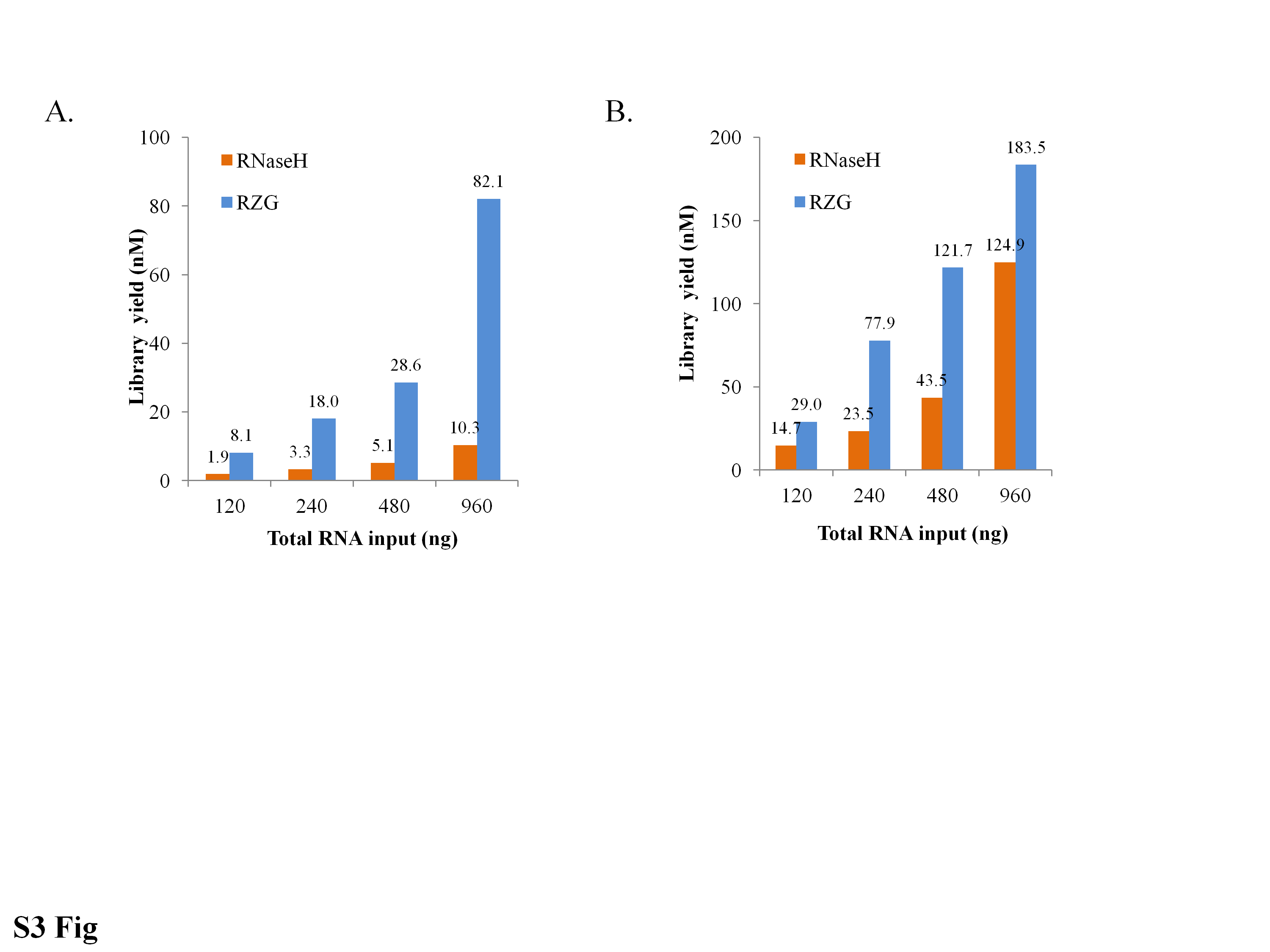

Supplement: S3 Fig — (TIF) [file pone.0224578.s003.TIF]

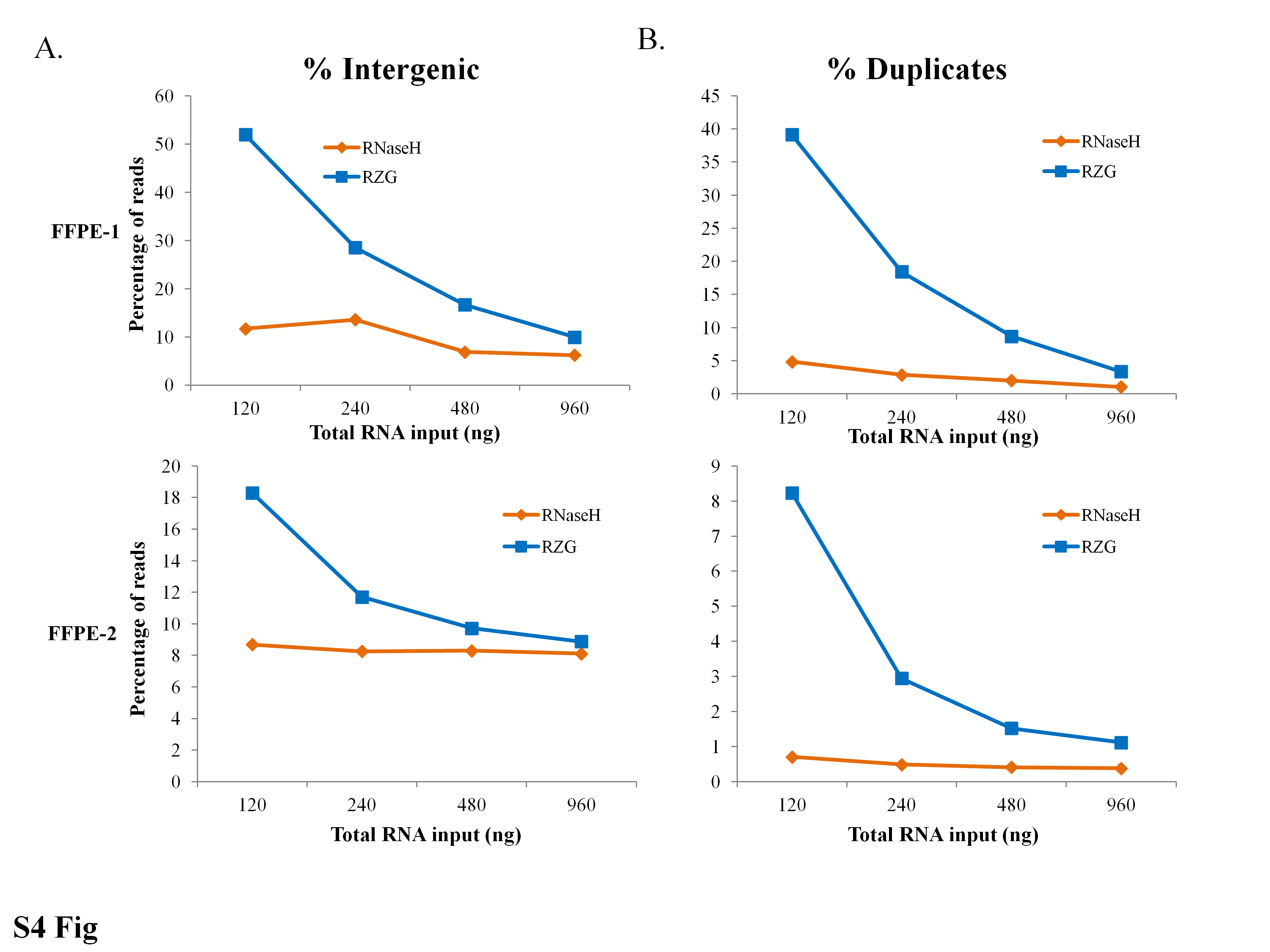

Supplement: S4 Fig — (TIF) [file pone.0224578.s004.TIF]

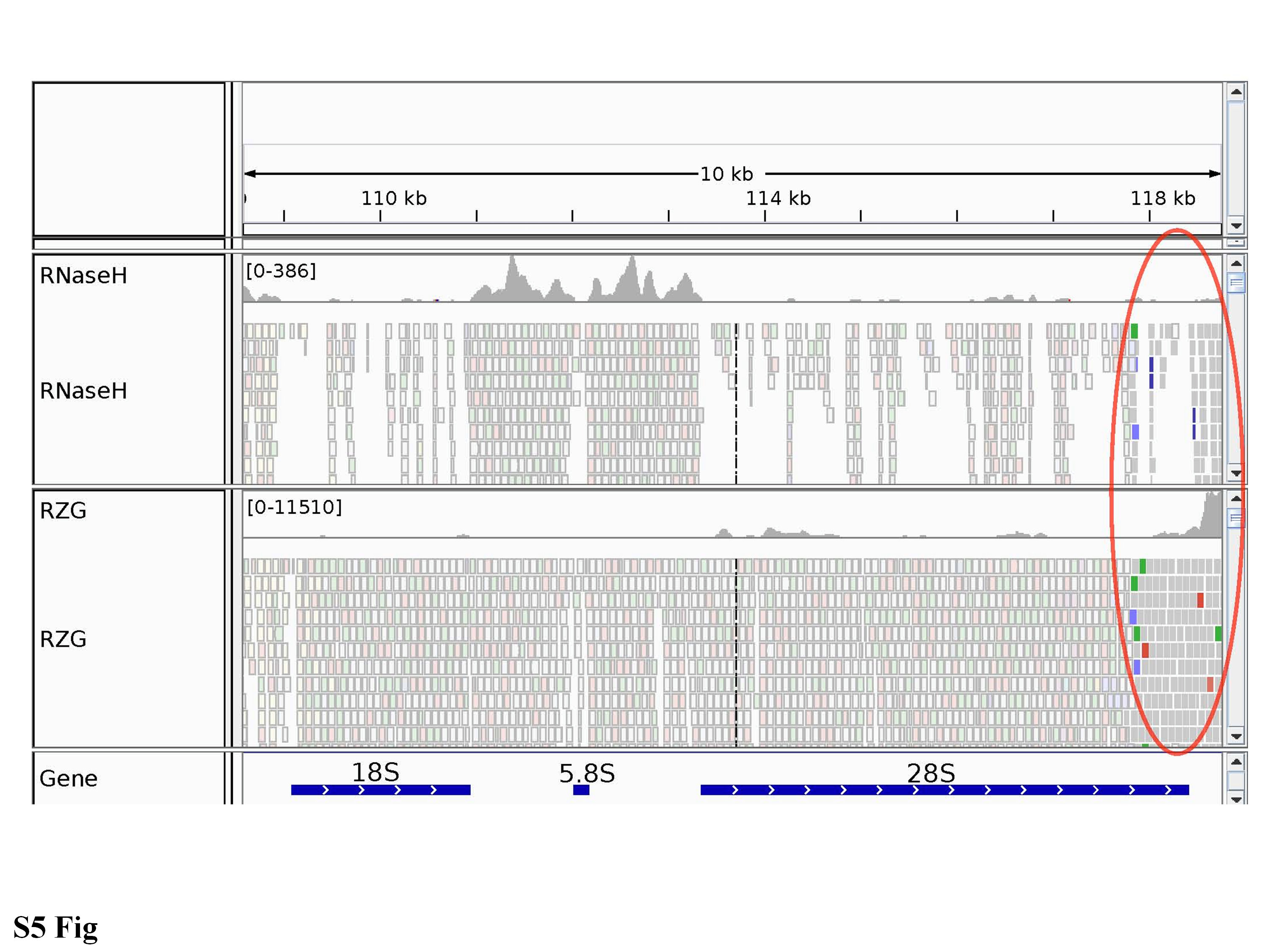

Supplement: S5 Fig — (TIF) [file pone.0224578.s005.TIF]

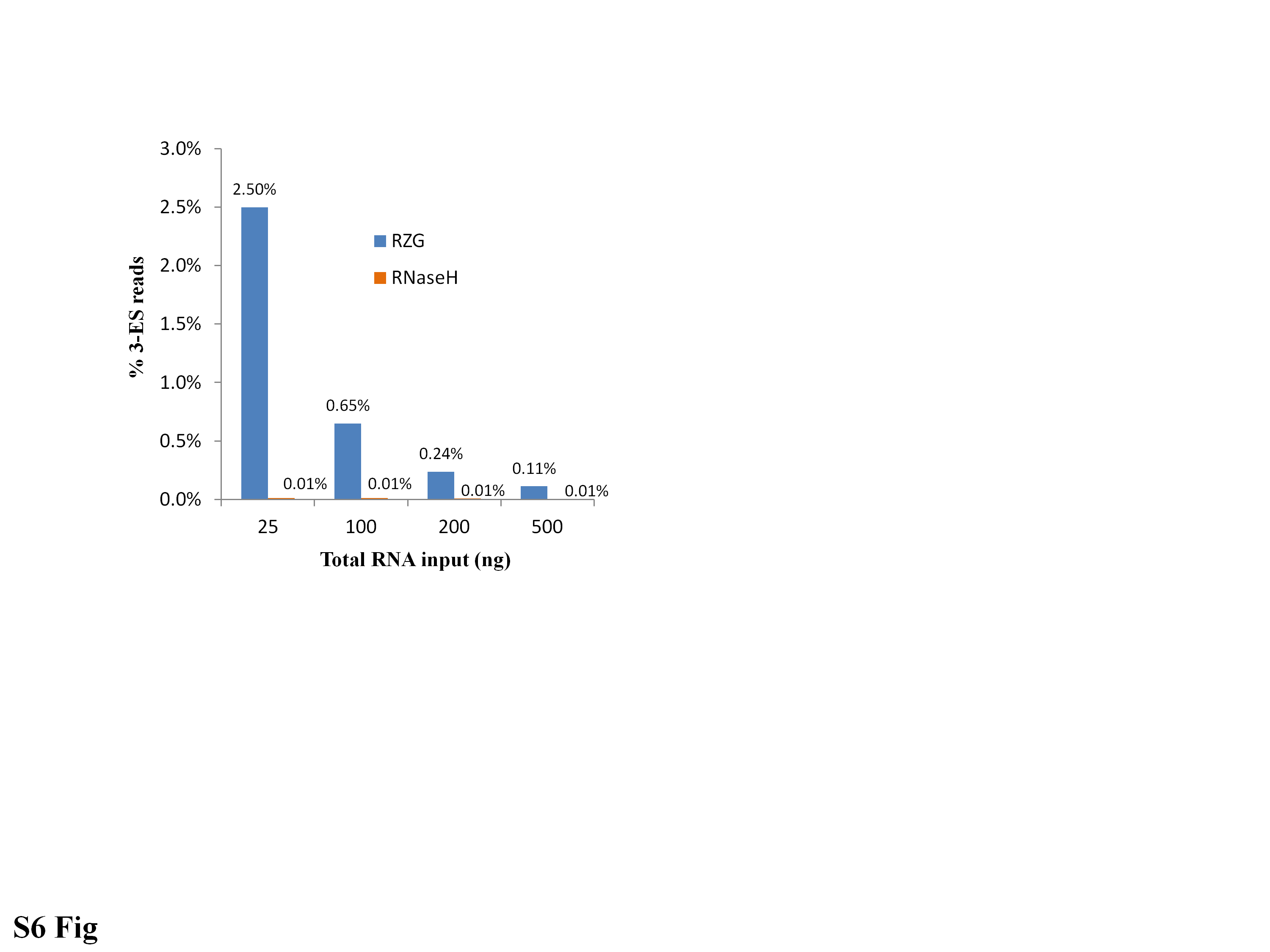

Supplement: S6 Fig — (TIF) [file pone.0224578.s006.TIF]

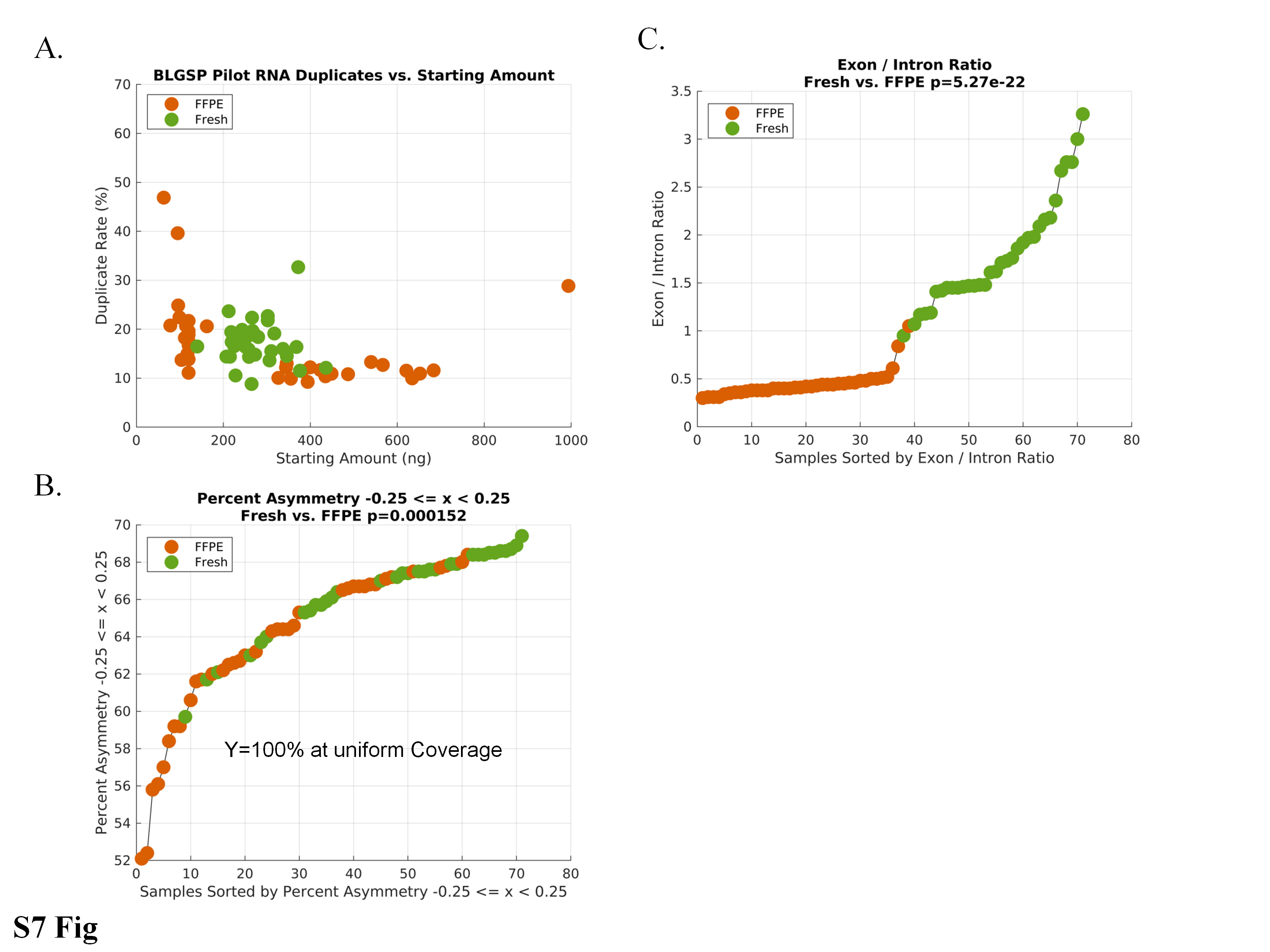

Supplement: S7 Fig — (TIF) [file pone.0224578.s007.TIF]
